# Functionalization of Silk with In-Situ Synthesized Platinum Nanoparticles

**DOI:** 10.3390/ma11101929

**Published:** 2018-10-10

**Authors:** Fan Zou, Ji Zhou, Jin Zhang, Jingliang Li, Bin Tang, Wu Chen, Jinfeng Wang, Xungai Wang

**Affiliations:** 1 National Engineering Laboratory for Advanced Yarn and Fabric Formation and Clean Production, Wuhan Textile University, Wuhan 430073, China; 15207136703@163.com (F.Z.); wuchen@wtu.edu.cn (W.C.); xungai.wang@deakin.edu.au (X.W.); 2 Hubei Collaborative Innovation Center for Advanced Organic Chemical Materials and Ministry-of-Education Key Laboratory for the Synthesis and Application of Organic Functional Molecules and College of Chemistry and Chemical Engineering, Hubei University, Wuhan 430062, China; zhouji@hubu.edu.cn; 3 Institute for Frontier Materials, Deakin University, Geelong, VIC 3216, Australia; jin.zhang@deakin.edu.au (J.Z.); jingliang.li@deakin.edu.au (J.L.)

**Keywords:** platinum nanoparticle, silk, coloration, catalysis, antibacterial

## Abstract

After platinum nanoparticles (PtNPs) were in-situ synthesized on silk fabrics through heat treatment, it was determined that the treatment of the silk fabrics with PtNPs imparted multiple functions, including coloring, catalysis, and antibacterial activity. The formation of PtNPs on fabrics was affected by the Pt ion concentration, pH value of solution, and reaction temperature. Acidic condition and high temperature were found to facilitate the formation of PtNPs on silk. The color strength of silk fabrics increased with the concentration of Pt ions. The PtNP treated silk fabrics exhibited reasonably good washing color fastness and excellent rubbing color fastness. The morphologies and chemical components of the treated silk fabrics were analyzed using scanning electron microscopy and X-ray photoelectron spectroscopy. The PtNP treated silk fabric exhibited significant catalytic function and a notable antibacterial effect against *Escherichia coli* (*E. coli*).

## 1. Introduction

Functional fabrics have attracted considerable research attention because of their broad applications in both industrial and daily life. Many strategies have been developed to coat fibrous materials with nanoparticles. For example, titanium dioxide (TiO_2_) and zinc oxide (ZnO) nanoparticles were used to modify the surface of fabrics to realize the UV blocking function of textile products [1,2,3]. Noble metal nanoparticles have also been used for the coloration and functionalization of fibers [4,5]. For example, gold and silver nanoparticles have strong absorption of photon energy at certain wavelengths, enabling fabrics coated with these nanoparticles to display bright colors, due to their localized surface plasmon resonance (LSPR) properties. Cotton fabrics functionalized with in-situ synthesized gold nanoparticles not only showed vivid color, but also possessed significant ultraviolet-blocking and antibacterial properties [6]. The ramie fibers modified with silver nanoparticles exhibited great catalytic activity in addition to bright color [7]. Besides, Wu et al. loaded platinum nanoparticles (PtNPs) on porous cellulose nanocrystals and studied the catalytic property of cellulose nano-composites [8]. Yun et al. obtained the textile materials coated with PtNPs after treating knitted cotton webs with tannic acid and ferric iron. The cotton webs with PtNPs exhibited strong catalytic activity and good recyclability [9].

Silk is an excellent natural fibrous material with notable gloss and softness, good moisture absorption, and breathability. Since silk is a type of protein, it is easy for microorganisms and bacteria to accumulate and proliferate on silk fibers in a humid environment [10,11], which can cause damage to fibers in the silk products and even bring about skin diseases. Many antibacterial agents have been applied to eliminate microbial damage to natural fibers [12,13]. Compared with organic reagents, inorganic nanomaterials, especially PtNPs, are stable and effective for antimicrobial applications [12]. PtNPs have high activity [14,15] and selectivity for catalytic reaction. They can enhance the cleansing function of skin surface. Meanwhile, PtNPs can retard the growth of *Escherichia coli* (*E. coli*) and *Staphylococcus aureus* (S. aureus) to achieve antibacterial effects [16]. However, PtNPs are prone to aggregation and precipitation, limiting effectiveness in intended applications. Recently, Aladpoosh and Montazer et al. attached Ag and ZnO to cotton fabric by heating the fabric in a water bath. The treated fabric showed good antibacterial activity [17]. Yang and co-workers successfully loaded PtNPs onto cotton, and the obtained cotton fabric showed a cyclic catalytic function [14]. 

Herein, we developed a convenient and feasible approach to accomplish functional surface modification of silk fabrics based on in-situ synthesis of PtNPs on silk by heat treatment. The PtNPs endowed the silk fabrics with colors, antibacterial property, and catalytic activity. The optical properties of the functionalized fabrics were observed using K/S curves and UV-vis reflectance spectroscopy. We systematically investigated the influences of Pt ion concentration, reaction temperature, and pH value on the optical features and catalytic performance of the functionalized silk fabric. The morphology and chemical components of the obtained silk fabrics were characterized. The catalytic activity and antibacterial property of the functionalized silk fabrics were evaluated. Moreover, the color fastness of the treated fabrics to washing and rubbing was assessed.

## 2. Materials and Methods

### 2.1. Materials

Chloroplatinic acid (H_2_PtCl_6_·6H_2_O, ≥37%), NaOH (≥96.0%), acetic acid (≥99.5%), 4-nitrophenol (4-NP) (≥99%), and sodium borohydride (NaBH_4_) (98%) were purchased from Aladdin (Shanghai, China). All chemicals were analytical grade reagents, and used without further purification. Woven silk fabrics (97 g·m^−2^) with 285 warps (per 5 cm) and 285 wefts (per 5 cm) were obtained from a local retailer. 

### 2.2. In-Situ Synthesis of PtNPs on Silk Fabrics

Silk fabrics were washed for 3 min using warm water (50 °C) and then rinsed with deionized water at room temperature. The washed fabrics were immersed in different concentrations (0.1, 0.2, 0.3, and 0.4 mM) of H_2_PtCl_6_ solutions with a 100:1 of weight ratio (aqueous solution to silk fabrics). The pH value of solutions was measured at 5. Silk fabrics were incubated in the H_2_PtCl_6_ solutions for 10 min. Then the solutions were heated at 90 °C for 60 min in a shaking water bath. The fabrics after treatment were rinsed with running deionized water and dried at room temperature. The samples corresponding to 0.1, 0.2, 0.3, and 0.4 mM of H_2_PtCl_6_ solutions were denoted by PSF1, PSF2, PSF3, and PSF4, respectively. In order to observe the influence of pH values, NaOH or acetic acid was added to H_2_PtCl_6_ solutions to adjust the pH values of reaction system from 3 to 6. In addition to 90 °C, other temperatures (85 °C, 95 °C, and 100 °C) were tried to investigate the impact of temperature on in-situ synthesis of PtNPs on silk fabrics.

### 2.3. Characterization Instrument

Scanning electron microscopy (SEM) measurements were carried out on a Hitachi SU8010 (Tokyo, Japan). X-ray photoelectron spectroscopy (XPS, Manchester, UK) analysis were implemented on a Kratos XSAM800 XPS system with Kα source and a charge neutralizer. Platinum content was measured by an IRIS Intrepid II XSP inductively coupling plasma atomic emission spectrometer (ICP-AES) instrument (Manchester, UK). An Ocean Optics USB4000 spectrometer (Dunedin, FL, USA) was used to record the UV-vis absorption spectra of solutions. UV-vis diffuse reflectance spectra of fabrics were obtained using BRC642E B&W Tek BRC642E CCD spectrometer (Newark, DE, USA) contented with an Ocean Optics reflection and backscattering fiber probe (Dunedin, FL, USA). A Bruker D8 Advance X-ray diffractometer with Cu Kα radiation (Madison, WI, USA) was used to obtain X-ray diffraction (XRD) patterns. The color strength (K/S) of specimens was calculated using the Kubelka–Munk equation as follows:

 KS=(1−R)22R 

where *K* and *S* are the absorption and scattering coefficients of fabrics, respectively. *R* is the reflectance of the fabric at maximum absorption, measured using an X-rite Color i7 spectrophotometer (Grand Rapids, MI, USA).

### 2.4. Color Fastness to Washing and Rubbing

Washing fastness was evaluated in accordance with the Australian Standard (AS 2001.4.15-2006). The silk fabrics treated with PtNPs were washed for 45 min at 50 °C in the presence of ECE reference detergent (4.0 g L^−1^) using a lab dyeing machine (Ahiba IR Pro, Datacolor International, Lawrenceville, NJ, USA). The CIE Lab color coordinate values (L*, a*, and b*) for specimens were measured before and after washing, using an X-rite Color i7 spectrophotometer (Grand Rapids, Michigan, MI, USA). L* denotes the lightness/darkness; a* denotes chromaticity coordinates for red/green; and b* denotes yellow/blue chroma. The color difference (ΔE) was assessed using the changes in color coordinates (ΔL*, Δa*, and Δb*) with the formula: ΔE = [(ΔL*)^2^ + (Δa*)^2^ + (Δb*)^2^]^1/2^. 

The rubbing colorfastness of treated silk fabrics was estimated in accordance with the Australian Standard AS 2001.4.3-1995. The fabrics colored with PtNPs were rubbed using an undyed cotton cloth. The staining of the cotton cloths was evaluated based on the standard grayscale for staining. Both dry and wet rubbing fastness tests were performed.

### 2.5. Catalytic Activity

To study the catalytic activity of the PtNP functionalized silk fabrics, the catalytic conversion of 4-NP into 4-aminophenol (4-AP) by NaBH_4_ was conducted with the pristine and treated fabrics. In a typical procedure, NaBH_4_ solution (1.0 mL, 3.42 M) was dropped into 4-nitrophenol aqueous solution (40 mL, 0.02 mM). After that, silk fabrics of 8.5 mg (pristine silk, PSF1, PSF2, PSF3, and PSF4) were immersed into the mixing solution of 4-NP and NaBH_4_ under vigorous stirring. UV-vis absorption spectroscopy (Ocean Optics USB4000 spectrophotometer, Largo, FL, USA) was employed to record the conversion of 4-NP into 4-AP to compare the catalytic performance of different silk fabrics. 

### 2.6. Antibacterial Test Against Gram-Negative Bacteria

Gram-negative bacteria, *E. coli* (ATCC 25922), were used as test organisms. Antibacterial tests were performed on both the pristine and the PtNP treated silk fabrics (PSF3), in accordance with the AATCC 100-2012 (Clause 10.2) standard with slight modifications. In brief, the bacteria (50 µL) were added to the samples in flasks, followed by pouring of 50 mL of sterile deionized water under vigorously shaking. The flasks were incubated for 24 h at 37 °C in an incubator shaker (Xiangyi Instrument Co., Ltd, Xiangtan, China). After that, the fabric specimens were collected and the solution left in the flask was further diluted to obtain counts of bacterial colonies. 100 µL of the 10^3^ dilution obtained was placed on the nutrient agar plates. The agar plates were then incubated for 24 h at 37 °C in an oven. The antibacterial activity of the fabric samples were analyzed by the quantitative method of counting microbial colony forming units (CFU) of *E. coli*. The percent reduction of the bacteria was calculated as follows:

Reduction in CFU (%) = (C − A)/A × 100%

where, C and A are the bacterial colonies for the control and fabric samples, respectively.

## 3. Results and Discussion

### 3.1. Preparation and Characterization of the Silk Fabrics with PtNPs

**Figure 1:**
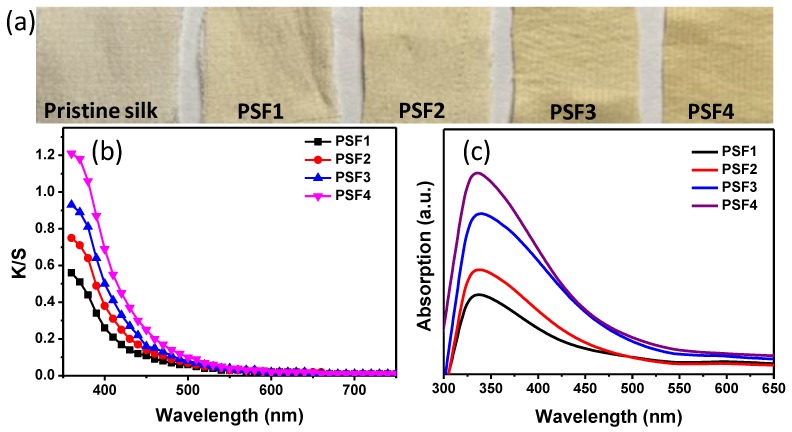
(**a**) Photograph, (**b**) K/S curves and (**c**) UV-vis diffuse reflective absorption spectra of the PtNP treated silk fabrics.

The color of silk fabrics changed from white to yellow after the PtNPs were in-situ synthesized onto its surface (Figure 1a). The fabrics treated with 0.1 mM of H_2_PtCl_6_ showed a slightly yellow color. The yellow color of silk fabrics deepened as the initial concentration of H_2_PtCl_6_ increased. The silk fabrics with 0.4 mM of H_2_PtCl_6_ presented a deeper color in yellow than in other fabrics. The color change of the treated silk fabrics may be attributed to the variation of morphology and loading percentage of PtNPs on silk fibers, similar to the case of coloration of cotton by gold nanoparticles in our previous work [6]. ICP-AES testing confirmed that the platinum contents of the treated silk fabrics were 3.50, 4.39, 8.58, and 9.37 mg g^−1^ corresponding to PSF1, PSF2, PSF3, and PSF4, respectively. The platinum content increased with the increase of the initial concentration of H_2_PtCl_6_ (Table 1).

The color strength (K/S) was further measured to gain insights into the color changes of silk fabrics (Figure 1b). The peaks of K/S curves of the treated silk fabrics were located at around 360 nm. The maximum K/S value increased as the platinum content increased on silk fabrics, which was consistent with the deepening trend of fabric color. To further observe the optical properties of the fabric specimens, UV-vis diffuse reflectance absorption spectra of PtNP treated silk fabrics were recorded. A single absorption band located at around 335 nm appeared in the UV-vis absorption spectrum of PSF1 (Figure 1c), which is ascribed to the characteristic LSPR mode of PtNPs [18,19,20]. The LSPR bands of the PtNP treated silk fabrics red-shifted from 335 nm to 339 nm when the initial concentration of H_2_PtCl_6_ increased from 0.1 mM to 0.3 mM, along with an increase in absorption intensity. When the H_2_PtCl_6_ concentration increased from 0.3 mM to 0.4 mM, the UV-vis absorption peak blue-shifted and the absorption intensity continued to increase. Combining the results of ICP-AES and UV-vis absorption spectroscopy, we can see that the intensity of UV-vis absorption bands increased with the content of PtNPs on silk. The changes of optical properties of the treated silk fabrics may be associated with the morphologies and coating density of PtNPs on the silk fabrics. 

**Figure 2:**
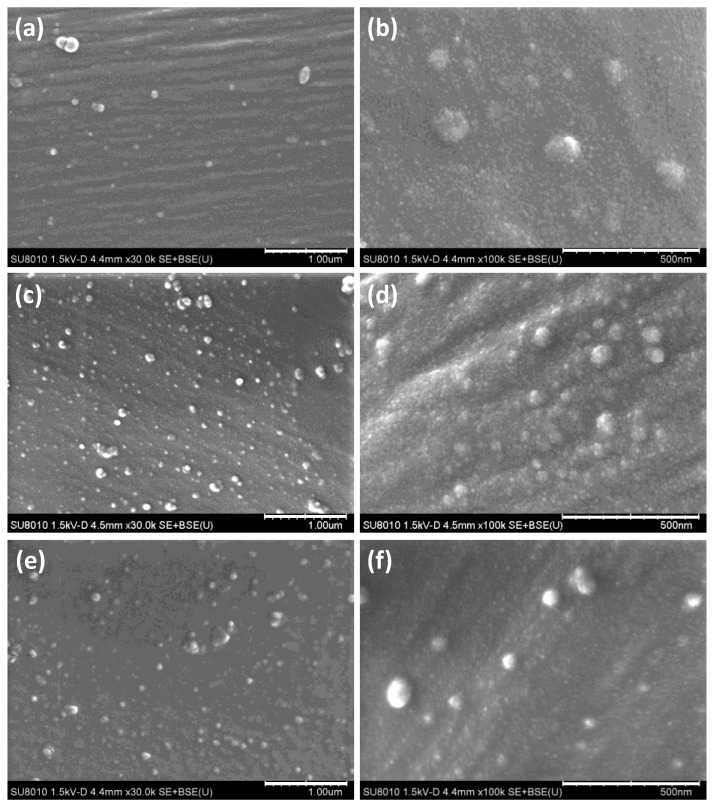
Scanning electron microscopy (SEM) images of the silk fabrics treated with different concentrations of Pt ions: (**a**,**b**) 0.2 mM (PSF2); (**c**,**d**) 0.3 mM (PSF3); (**e**,**f**) 0.4 mM (PSF4).

The surface morphologies of the fabric samples were observed using SEM, as shown in Figure 2. A large number of nanoparticles were evenly coated on the fiber surface, indicating that PtNPs were in-situ synthesized onto silk fabrics. For PSF2, both the large particles with sizes of 81.4 ± 31.9 nm and the small nanoparticles with size of 20.6 ± 8.0 nm (Figure 2a,b) were found on the fiber surface. The coating density of nanoparticle on the silk fiber surface increased as the concentration of H_2_PtCl_6_ increased from 0.2 to 0.3 mM (Figure 2c,d). Meanwhile, there was an obvious size increase for the small particles, which increased from a size of 20.6 ± 8.0 nm for PSF2 to 23.2 ± 8.9 nm for PSF3. The size of the small nanoparticles remained unchanged nearly as the H_2_PtCl_6_ concentration further increased to 0.4 mM (22.6 ± 7.5 nm). Changes in nanoparticle size resulted in different UV-vis absorption results of PtNP treated silk fabrics. 

**Figure 3:**
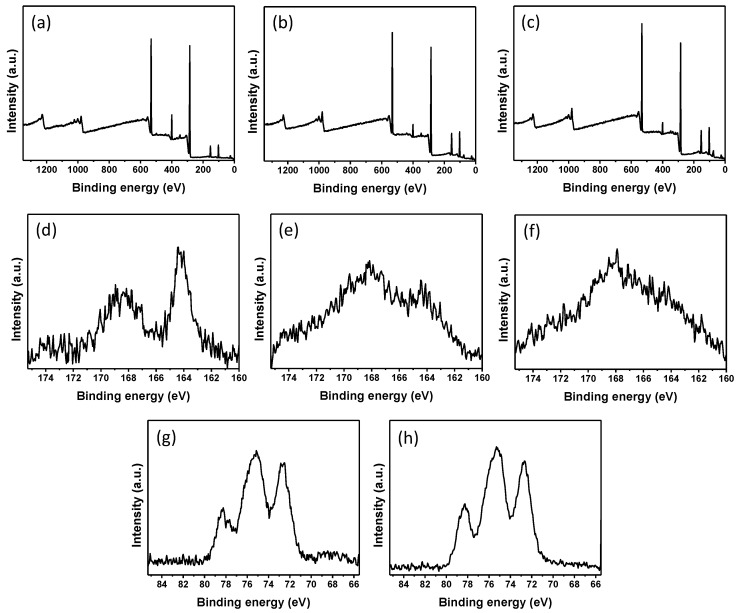
X-ray photoelectron spectroscopy (XPS) spectra of (**a**) the pristine silk fabric; (**b**) PSF2 and (**c**) PSF4. S 2p XPS spectra of (**d**) the pristine silk fabric; (**e**) PSF2 and (**f**) PSF4. Pt 4f XPS spectra of (**g**) PSF2 and (**h**) PSF4.

**Figure 4:**
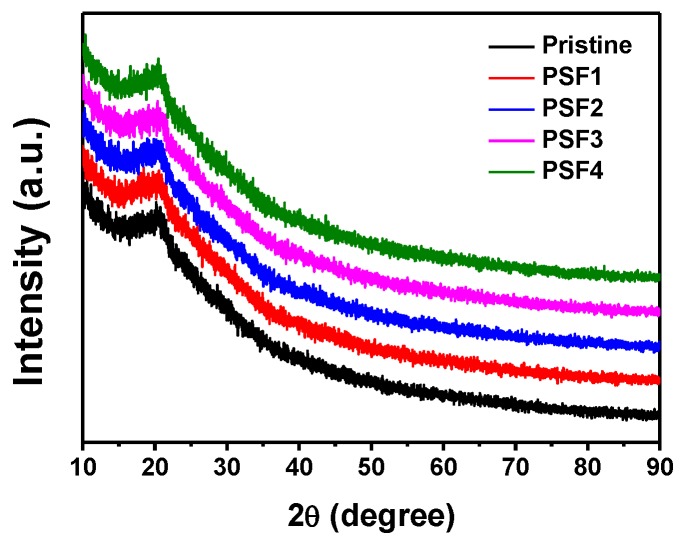
X-ray diffraction (XRD) patterns of different fabric samples.

XPS was employed to analyze the fabric surface (Figure 3). The characteristic peaks which were assigned to (O 1s), (C 1s), (N 1s), and (S 2p) as the normal components of the silk were observed in the XPS spectra of the pristine silk (Figure 3a) and the treated silk fabrics (PSF2 and PSF4) (Figure 3b,c). For the S 2p spectrum of the pristine silk, a 164.2 eV has been seen (Figure 3c), which may be ascribed to the signal of sulfur of the disulfide bonds in silk cystine residues [21,22,23,24]. Other peaks at 168.4 eV assigned to the oxidized species of S from the cysteic acid was also found (Figure 3c). Whereas, the XPS peaks at 164.2 eV corresponding to disulfide bonds nearly disappeared after the silk fabrics were treated (Figure 3e,f), implying that the disulfide bonds were broken, which may result from the heat treatment at high temperature. The remained XPS peak at 168.4 eV indicated that oxidized species of S were present on the surface of the treated silk. The peaks at 78.2 eV and 75.1 eV which were assigned to the oxidized species of Pt (Pt (IV)) were observed in the XPS spectrum of PSF2 and PSF4 (Figure 3g,h) [25], revealing that Pt (IV) existed on the surface of the silk fabrics. Another peak at 72.7 eV also arose in the XPS spectra of the treated silk. The XPS peak could be assigned to the Pt (II) species [26,27,28]. The binding energy of metallic platinum is located around 71.0 eV [11,27,29]. The interaction between surface platinum atoms and sulfur atom in cysteine on silk led to higher energy in comparison with pure metallic platinum [11,30]. XPS data suggests that the charge transfer between platinum and sulfur could occur. The linking of Pt-S could improve the durability of Pt NPs on silk fabrics. Moreover, no notable new XRD peaks appeared after the fabrics were treated with PtNPs (Figure 4). This may be due to the limited amount and low crystallization ratio of PtNPs formed on the silk fabrics.

### 3.2. Influence of pH Value and Temperature

**Figure 5:**
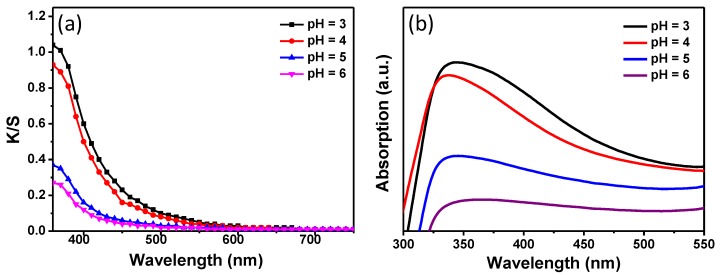
(**a**) K/S curves and (**b**) UV-vis diffuse reflectance absorption spectra of silk fabrics treated in the presence of 0.3 mM of H_2_PtCl_6_ at different pH values.

**Figure 6:**
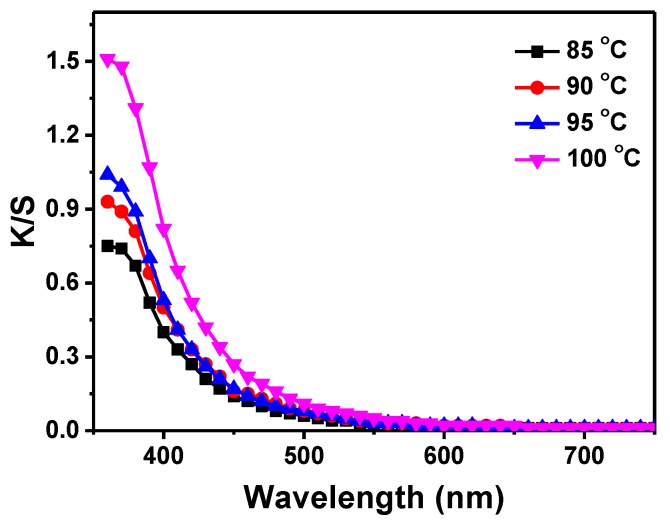
K/S curves of the silk fabric treated at different temperatures with 0.3 mM of H_2_PtCl_6_.

The fabric samples were treated with 0.3 mM of H_2_PtCl_6_ solution at different pH values (3~6) to study the pH effect on in-situ synthesis of PtNPs on silk fabrics. The K/S curves and UV-vis diffuse reflectance absorption spectra are shown in Figure 5. The largest K/S value of 1.04 was obtained when the fabric was treated at pH = 3 (Figure 5a). The maximum K/S value of the silk fabrics decreased with increasing pH values of the reaction solution and it decreased to 0.27 when the pH value increased to 6. Bright colors of silk fabrics were achieved when the pH was less than 5. The color of the silk fabrics showed negligible change when the pH value was above 7, revealing that almost no PtNPs formed on the silk fabrics under this condition. Figure 5b displays the UV-vis reflectance absorption bands of the silk fabrics treated with PtNPs at different pH values. The fabric samples prepared at pH = 3 and 4 exhibited pronounced UV-vis absorption peaks with intensities higher than those prepared at pH = 5. The intensity increase of the UV-vis absorption bands at low pH conditions was consistent with that of the K/S values. In addition, the platinum content of the fabrics enhanced as the pH value reduced from 6 to 3 (Table 2). The results demonstrated that the acidic condition facilitates the in-situ synthesis of PtNPs on silk fabrics. Moreover, K/S values of silk fabrics were measured at different treatment temperatures to investigate the effect of temperature on the in-situ synthesis of PtNPs (Figure 6). The maximum K/S values of the treated silk fabrics increased when the reaction temperature changed from 85 to 100 °C, which reveals that higher temperature assists with fabrication of more PtNPs on silk fabrics.

### 3.3. Assessment of Color Fastness 

**Figure 7:**
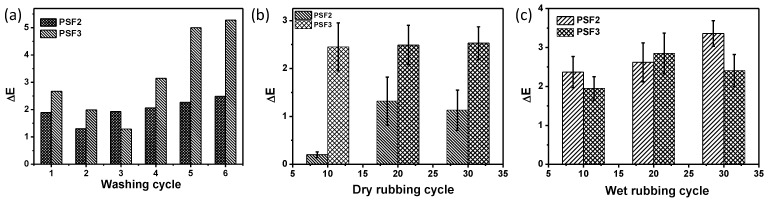
Evolution of color difference (ΔE) of the treated silk with PtNPs (PSF2 and PSF3) with (**a**) washing cycles, (**b**) dry rubbing cycles and (**c**) wet rubbing cycles.

The durability of PtNPs on silk fabrics can be evaluated by measuring the color fastness of silk fabrics. Color fastness is an important parameter that affects the practical performance and usage of textile products. The color fastness to washing cycles of the PtNP coated silk fabrics was tested by washing the fabric samples with the ECE reference detergent for 45 min at 50 °C, for each washing cycle. The color difference (∆E) values of the fabrics before and after washing are shown in Figure 7a. After the first washing cycle, the ∆E values were 1.9 and 2.7 corresponding to PSF2 and PSF3, respectively, which reveals that colors of the fabrics faded during washing. The ΔE of PSF2 maintained to be around 2.3 with the washing cycles, which demonstrates its good colorfastness to washing for PSF2. The ΔE values of PSF3 changed slightly after five washing cycles. The PtNP coated silk fabrics exhibited reasonably good color fastness to washing. We additionally tested the color fastness of the treated silk fabrics to rubbing. The grayscale rating for color differences of PSF2 and PSF3 under dry and wet rubbing conditions was evaluated. The color fastness was rated as 5 for both PSF2 and PSF3, after 20 dry rubbing cycles. Moreover, the grayscale was rated as 5 and 4–5 for PSF2 and PSF3, respectively, even after 20 wet rubbing cycles. Figure 7b,c display the ΔE values of PSF2 and PSF3, during dry and wet rubbing. It can be seen that the ΔE values of PtNP treated silk fabrics changed slightly after 20 cycles of dry or wet rubbing. These results indicate that the PtNP treated silk fabrics have very good color fastness to rubbing.

### 3.4. Investigation of Catalytic Activity

**Figure 8:**
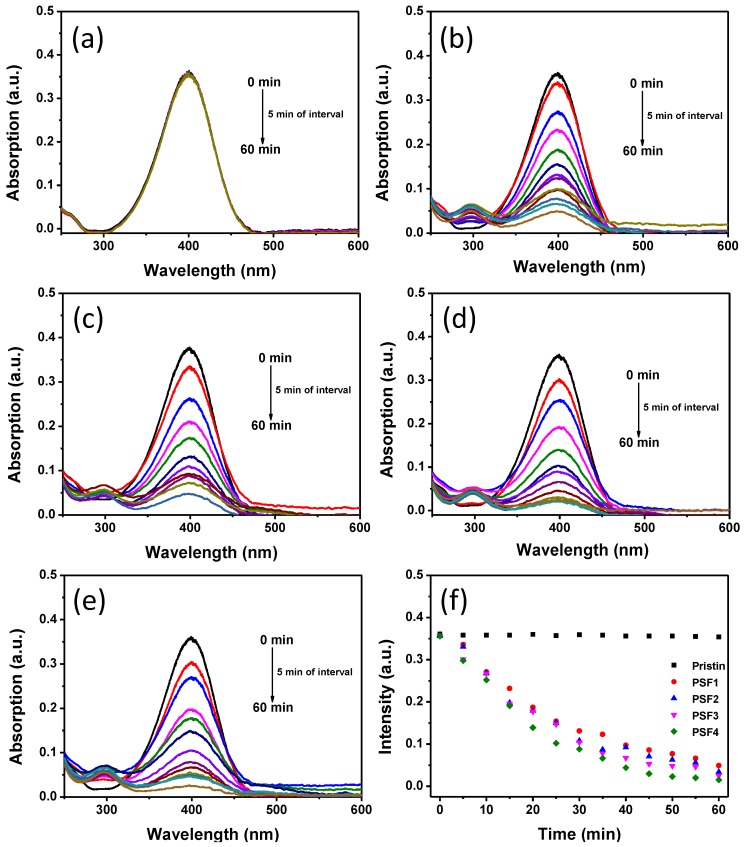
Evolution of UV-vis absorption spectra of 4-nitrophenol solution after NaBH_4_ solution in the presence of the different fabrics: (**a**) pristine fabric; (**b**) PSF1; (**c**) PSF2; (**d**) PSF3; and (**e**) PSF4. (**f**) Plots of the peak intensity as a function of reaction time in the presence of different silk fabrics.

PtNPs as common catalysts have been extensively used to accelerate reactions. In this study, PtNPs were attached to silk fibers by the in-situ synthesis process. Silk fabrics can act as a supporting substrate for the nanomaterials, which facilitates with the separation of PtNPs from the reaction system for reuse in the next reaction cycle. The reduction of 4-NP by NaBH_4_ is widely used as a catalytic model reaction to analyze the catalytic activity of metal nanoparticle catalysts [31]. The UV-vis absorption spectra of aqueous solution during the reduction of 4-NP were recorded to assess the catalytic ability of the PtNP treated silk fabrics. The 4-NP solution changed from light-yellow to green-yellow when NaBH_4_ was added into the reaction system. The reduction of nitro compounds by NaBH_4_ is very slow without any catalyst in the solution, whereas metal nanoparticles can accelerate the reduction reaction through transferring electron from NaBH_4_ to the nitro compounds [32]. The formation of 4-nitrophenolate ions resulted in a new UV-vis absorption peak at 400 nm after the addition of NaBH_4_ [31]. The UV-vis absorption spectra of the solution mixture of 4-NP and NaBH_4_ changed slightly in the presence of pristine silk fabrics (Figure 8a). No visible decrease of the 4-NP absorption peak at 400 nm was observed within 60 min, which reveals that the pristine silk fabric has no catalytic activity. Figure 8b–e shows the evolution of the UV-vis absorption spectra of the 4-NP solution in the presence of PtNP treated cotton fabrics (PSF1–PSF4) after the addition of NaBH_4_. The intensity of absorption band at 400 nm dropped remarkably for all the PtNP treated silk fabrics. Meanwhile, a new absorption peak at 300 nm appeared during the reduction process of 4-NP, which is attributed to the generation of 4-AP [33,34]. The reduction rate of 4-NP can be indicated by the plots of the intensity of the 400 nm band versus time (Figure 8f). The peak intensity at 400 nm of the 4-NP solution with the PtNP treated silk fabrics immensely reduced, implying that the PtNP treated silk fabrics possess great catalytic activity. The PtNP loading imparted catalytic feature to silk fabrics, which may facilitate the degradation of organic contaminants on the silk clothes in real life. 

**Figure 9:**
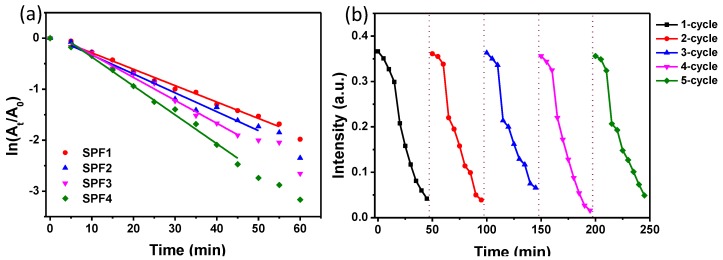
(**a**) Plots of n(A_t_/A_0_) at 400 nm as a function of reaction time in the presence of different silk fabrics. (**b**) Recycling and reuse of the PtNP treated silk fabric (PSF3) for the reduction of 4-NP to 4-AP.

Generally, the reduction of 4-NP into 4-AP is considered as a pseudo-first-order kinetic reaction in the presence of adequate NaBH_4_ [35,36]. This pseudo-first-order hypothesis for reduction of 4-NP was demonstrated by the linear correlation between ln(A_t_/A_0_) and time (Figure 9a). The apparent rate constant (K_app_) of the catalytic reaction can be calculated based on the linear slope of ln(A_t_/A_0_) versus time. The K_app_ value of the reduction reaction was estimated to be 3.22 × 10^−2^, 3.70 × 10^−2^, 4.49 × 10^−2^ and 5.67 × 10^−2^ min^−1^ for PSF1, PSF2, PSF3, and PSF4, respectively. The Kapp values obtained in this work are comparable to Islam et al.’s work reported for the PtNPs [37]. The Kapp for the treated silk fabric enhanced with the increase of the amount of PtNPs on the surface of silk fabrics, indicating that the catalytic activity is determined by the content of platinum on the silk fabrics. In order to evaluate the durability of the catalyst, the treated fabric (PSF3) was separated from the reaction solution and reused in the repeated reduction system of 4-NP. The peak intensity at 400 nm for each conversion cycle was plotted as a function of time as shown in Figure 9b. The treated fabric maintained great catalytic activity even after five cycles, revealing that the PtNP treated silk fabrics presented durable catalytic feature.

### 3.5. Antibacterial Properties

**Figure 10:**
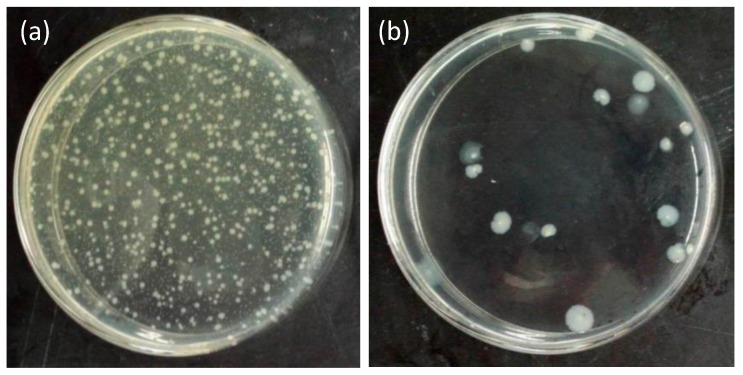
Evaluation of the antibacterial activity of (**a**) the pristine silk fabric and (**b**) the PtNP treated silk fabric (PSF3).

The antibacterial activity of PtNPs or Pt(IV) ion has been investigated in the previous reports [18,38,39]. In this study, we evaluated the antibacterial property of the PtNP treated silk fabrics against *E. coli.* Figure 10 shows the bacteria colonies on the agar plats for the pristine and treated fabrics. The plates for the pristine fabric had bacteria fully covered (Figure 10a), whereas few bacteria colonies were seen on the agar medium of the PSF3. Antibacterial properties of fabric samples were further evaluated by reduction of CFU. The reduction of CFU corresponding to pristine and treated fabrics was 0% and 91%, respectively. These results suggest that the PtNPs or Pt(IV) ions on the silk fabrics distinctively hindered the growth of bacteria (Figure 10b). The phenomena proved that the PtNP-treated silk fabrics possessed remarkable antibacterial activity.

## 4. Conclusions

A facile method has been developed to in-situ PtNPs on silk fabric by the reduction of Pt ions using a heat treatment. The surface modification with PtNPs endowed silk fabric with colors, catalysis, and antibacterial characteristics. The color strength increased with the Pt ion concentration. Low pH value and high temperature were conducive to the in-situ formation of PtNPs on silk fabrics. The PtNP-treated silk fabrics exhibited reasonably good color fastness to washing and excellent color fastness to rubbing. More importantly, the PtNP modified fabrics showed great catalytic and antibacterial activities. The as-prepared PtNP-treated silk fabrics have potential applications in functional textile products and ornaments. 

## Figures and Tables

**Table 1:** Pt content of silk fabrics treated in the presence of different concentrations of H_2_PtCl_6_.

Sample ID	PSF1	PSF2	PSF3	PSF4
**Pt Content (mg g^−1^)**	3.50	4.39	8.58	9.37

**Table 2:** Pt content of silk fabrics treated at different pH values with 0.3 mM of H_2_PtCl_6_ at 90 °C.

**pH**	3	4	5	6
**Pt Content (mg g^−1^)**	8.60	8.58	1.81	0.68
